# Binding of Alpha-Crystallin to Cortical and Nuclear Lens Lipid Membranes Derived from a Single Lens

**DOI:** 10.3390/ijms231911295

**Published:** 2022-09-25

**Authors:** Raju Timsina, Samantha Wellisch, Dieter Haemmerle, Laxman Mainali

**Affiliations:** 1 Department of Physics, Boise State University, Boise, ID 83725, USA; 2 Biomolecular Sciences Graduate Programs, Boise State University, Boise, ID 83725, USA

**Keywords:** α-crystallin, single lens, cortical membrane (CM), nuclear membrane (NM), percentage of membrane surface occupied (MSO), binding affinity (K_a_), mobility parameter, maximum splitting, hydrophobicity, cataracts

## Abstract

Several studies reported that α-crystallin concentrations in the eye lens cytoplasm decrease with a corresponding increase in membrane-bound α-crystallin with age and cataracts. The influence of the lipid and cholesterol composition difference between cortical membrane (CM) and nuclear membrane (NM) on α-crystallin binding to membranes is still unclear. This study uses the electron paramagnetic resonance (EPR) spin-labeling method to investigate the α-crystallin binding to bovine CM and NM derived from the total lipids extracted from a single lens. Compared to CMs, NMs have a higher percentage of membrane surface occupied by α-crystallin and binding affinity, correlating with less mobility and more order below and on the surface of NMs. α-Crystallin binding to CM and NM decreases mobility with no significant change in order and hydrophobicity below and on the surface of membranes. Our results suggest that α-crystallin mainly binds on the surface of bovine CM and NM and such surface binding of α-crystallin to membranes in clear and young lenses may play a beneficial role in membrane stability. However, with decreased cholesterol content within the CM, which mimics the decreased cholesterol content in the cataractous lens membrane, α-crystallin binding increases the hydrophobicity below the membrane surface, indicating that α-crystallin binding forms a hydrophobic barrier for the passage of polar molecules, supporting the barrier hypothesis in developing cataracts.

## 1. Introduction

The continuously growing eye lens is divided into the cortical and nuclear regions depending on the age of the lens fibers in a specific layer [1,2]. The lens epithelium creates new fibers on top of previous fibers on the outer cortex. The organelles and nuclei in the initial fibers disappear, leaving mature fiber cells with only the membrane and cytoskeleton as their supramolecular structure [3,4]. Older fibers in the inner layers are known as the nucleus [1,2]. The lens fiber cells consist of a high concentration of structural proteins, namely α-, β- and γ-crystallins [5,6,7], which mainly function to maintain the transparency and refractivity of the lens [8]. α-Crystallin, which makes up ~40% of the lens protein [5,7], works as a molecular chaperone and a heat shock protein [9,10]. Since there is no turnover of proteins in the lens [11], the long-term transparency of the lens is highly dependent on the ability of α-crystallin to prevent the aggregation of denatured proteins and increase the tolerance to stress [12]. β- and γ-Crystallins, on the other hand, maintain the structure and refractivity of the lens [13]. 

α-Crystallin undergoes various mutations [14,15,16,17] and post-translational modifications [18,19,20,21,22] with aging, reducing its chaperone-like activity [23,24,25,26,27,28,29]. Most of the free α-crystallin in the human lens has been used up by the age of 40 years [30,31,32], following the significant increase in the level of water-insoluble and higher molecular weight complexes (HMWC) [33,34,35,36]. Some HMWC may bind with membranes and others may form in the cytoplasm distant from membranes [37]. It is still unclear how HMWC originate, together with light scattering and cataract formation [38,39,40]. A key mechanism for insolubilization of crystallins in aging lenses is believed to be the binding of crystallins to lens fiber cell membranes [37,41,42]. Mostly, all water-insoluble crystallins in the aged human lens bind to the membranes [42]. As the lens ages, large-scale binding of α-crystallin to lens membranes has been observed [38,39,41], which is highly believed to contribute to the onset of nuclear cataracts by occluding membrane pores and creating a barrier to diffusion [41,43,44]. A clinical investigation also shows that levels of α-crystallin in the eye lens cytoplasm decrease with a corresponding increase in the membrane-bound α-crystallin with age and cataracts [45]. Despite various studies on α-crystallin binding to lens membranes [37,40,46,47,48,49,50,51,52,53,54,55,56,57,58] and PL vesicles [46,57,59,60], the mechanism of α-crystallin binding to lens membranes is still unclear. Since the lipid (phospholipids (PLs) and sphingolipids (SLs)) and cholesterol (Chol) composition in the lens membranes differs dramatically among species [61,62,63,64,65], with locations in the lens [63,66,67,68,69,70,71,72] and with age and cataract formation [61,62,68,70,72,73,74,75,76,77], the role of changes in the lipid and Chol composition on α-crystallin binding to membranes needs further attention. This lipid and Chol composition changes may influence the α-crystallin’s binding to lens membranes, possibly causing cataracts [61]. 

For lens membranes containing proteins, the binding capacity of αA- and αB-crystallins to human cortical membrane (CM) and nuclear membrane (NM) is higher than for bovine CMs [58]. In addition, human NMs have higher binding capacities than CMs [58]. Previous studies of α-crystallin binding to native lens lipid membranes present conflicting results. Cobb and Petrash et al. [57] showed non-saturable binding of α-crystallin with synthetic and bovine cortical lens lipid vesicles, with no significant difference in binding of α-crystallin to all vesicles. However, Borchman and Tang et al. [46] reported saturable binding of α-crystallin to bovine lens lipid membranes and such binding made the headgroup regions of membranes less mobile. α-Crystallin binding could protect and stabilize the lipid bilayer and decrease membrane permeability [46]. Cobb and Petrash et al. [55] reported that the αA- and αB-crystallin homopolymers and αA: αB (3:1) heteropolymer bind to bovine lens membranes in a time- and temperature-sensitive manner. The binding of α-crystallin to membranes increases in acidic pH and upon removing exposed intrinsic protein domains but was not affected by high ionic strength, representing the hydrophobic binding of α-crystallin to membranes [55]. According to Bloemendal et al. [78], crystallins (primarily α-crystallin) bind significantly to the bovine lens membrane-cytoskeleton complex. Cenedella and Chandrasekher [79] reported that bovine lens lipid membranes have a high capacity for α-crystallin binding and such binding could be affected by the intrinsic membrane proteins. Ifeanyi et al. [59] demonstrated that phosphatidylcholine (PC) vesicles bound a maximum of 31.3 g of protein per mole of PL. Using lipids extracted from the cortical tissues of multiple bovine eyes, Sato et al. [54] demonstrated five times more α-crystallin binding to bovine CM than the PC vesicles reported by Ifeanyi et al. [59]. However, Cobb and Petrash et al. [57] observed no significant difference in the binding of αA-crystallin to bovine CM made of lens lipid and synthetic membranes made of sphingomyelin/cholesterol (SM/Chol). Liang and Li et al. [56] reported that, among α-, β- and γ-crystallins, only α-crystallin binds to bovine lens lipid membranes and lipid bilayers become less mobile with α-crystallin binding. Tang and Borchman et al. [40] showed that native bovine α-crystallin binds to bovine CMs made of lens lipid and the binding was closer on the surface of CMs at a higher temperature, probably due to increased exposure of hydrophobic surfaces of α-crystallin.

The lipid and Chol composition of the CM and NM differ significantly [63,66,67,68,69,70,71,72], with the NM having a higher Chol and SM content and a lower PC content than the CM [69]. Moreover, lens membrane SLs are roughly three to four times more saturated than PLs [74,80,81]. To the best of our knowledge, previous studies of α-crystallin binding to lens lipid membranes were performed either with only CMs [40,54,55,57] or with total lens lipid extracted from the whole lens [46,56,78,79]. Moreover, the majority of previous studies investigating α-crystallin binding to native lens lipid membranes include intrinsic membrane proteins [55,56,79], such as MIP26 and cytoskeletal proteins [58,78]. As far as we are aware, α-crystallin binding to CM and NM separately, in the absence of proteins within the membranes, has not been reported earlier. Therefore, it is essential to study the binding of α-crystallin to CM and NM made of lens lipid without proteins.

Previously, we successfully applied the electron paramagnetic resonance (EPR) spin-labeling methods to study the binding of α-crystallin to model membranes made of synthetic lipids. First, we studied the α-crystallin binding to individual and two-component PLs and SM membranes [82,83], even with different chain lengths and degrees of saturation of PLs [84]. Second, we examined the α-crystallin binding to cholesterol-containing individual PLs and SM membranes [85] and the cholesterol-containing model of human and animal lens lipid membranes [86]. Our previous studies [82,83,84,85,86,87] show that acyl chain length, degree of acyl chain unsaturation, lipid headgroups, lipid curvature and lipid and Chol composition strongly modulate α-crystallin binding to membranes and the physical properties of membranes. Building on our previous studies, we use the EPR spin-labeling method to study the α-crystallin binding to the bovine CM and NM derived from the total lipids extracted from a single lens of a two-year-old bovine. Unlike previous studies, the study reported in this paper focuses on the influence of lipid compositional differences in the bovine CM and NM made of lens lipid derived from the single lens on α-crystallin binding to these membranes. The Chol content in the human lens membrane decreases with cataracts [76,77]. In our study, Chol content in the CM has been decreased, mimicking the decreased Chol content in the cataractous lens membrane and α-crystallin binding to the cortical membrane with low cholesterol (CMLC) has also been studied. In the case of bovine lenses, large amounts of similar lenses (the main criterion is age) can be obtained from a meat packing plant. However, human lenses are more precious and difficult to obtain. Moreover, human lenses can be different not only because of age but also because of the varying health history of the donor. Even more significant is that a human’s right and left lenses may differ, as one eye may have a cataract and another may not. Therefore, the study reported in this manuscript is very significant because it shows the feasibility of performing α-crystallin binding to the CM and NM made of total lens lipid derived from a single lens of a human. 

## 2. Results

### 2.1. α-Crystallin Binding to the Cortical and Nuclear Membranes Derived from the Total Lipids Isolated from a Single Lens of a Bovine

**Figure 1:**
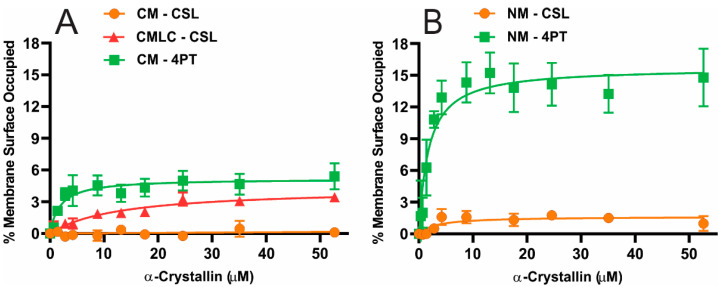
(**A**) The percentage of membrane surface occupied (MSO) by α-crystallin plotted as a function of α-crystallin concentration for the bovine CMs with the cholesterol analog spin-label (CSL) and 4-palmitamido-TEMPO (4PT) spin-label within these membranes and the CMLC with CSL spin-label within the membrane. (**B**) The MSO by α-crystallin plotted as a function of α-crystallin concentration for the bovine NMs with the CSL and 4PT spin-labels within these membranes. The CM and NM were derived from the total lipids extracted from a single lens cortex and nucleus of a two-year-old bovine. The Chol content in the CM was decreased by adding lipids resembling bovine lens lipid composition [63] to prepare the CMLC, as described in Section 4.3. The nitroxide moiety of the 4PT spin-label on the membrane surface (in the aqueous phase close to the membrane surface [88]) detected more MSO for both the CM and NM than the nitroxide moiety of the CSL spin-label below the membrane surface (near the headgroup regions [68,83,89]). The CSL spin-label does not detect α-crystallin binding to the CM; however, it detects the α-crystallin binding to the CMLC. The difference between the MMSO for these membranes with CSL and 4PT spin-labels is statistically significant with *p* ≤ 0.05. The bovine CM, the CMLC and the NM, with 11.4 mM total lipids, were incubated with varying concentrations of α-crystallin (0–52.6 μM) for 16 h at 37 °C and the EPR measurements were recorded at 37 °C. Results are the mean ± standard deviation (σ) from at least three independent experiments.

With increased α-crystallin concentration, the percentage of membrane surface occupied (MSO) by α-crystallin for CM with cholesterol analog cholestane spin-label (CSL) within the membrane remains zero, as shown in Figure 1A. This means that the CSL spin-label did not detect α-crystallin binding to the CM. Interestingly, the MSO by α-crystallin for the CMLC with CSL spin-label and the CM with 4-palmitamido-TEMPO (4PT) spin-label increases initially and remains saturated with the maximum percentage of membrane surface occupied (MMSO) of ~3.4 and ~5.4, respectively, representing the saturable binding of α-crystallin to these membranes, as shown in Figure 1A. With increased α-crystallin concentration, the MSO by α-crystallin for NM with CSL and 4PT spin-labels within the membrane increases initially and remains saturated, showing saturable binding of α-crystallin to the NMs, as shown in Figure 1B. The MMSO by α-crystallin for the NM with CSL and 4PT spin-labels is ~1.6 and ~15, respectively. The difference between the MMSO for all these membranes with CSL and 4PT spin-labels is statistically significant with *p* ≤ 0.05. For both the CM and NM, the nitroxide moiety of the 4PT spin-label on the surface of membranes (in the aqueous phase close to the membrane surface [88]) detected more of a α-crystallin binding (i.e., higher MMSO) than the nitroxide moiety of the CSL spin-label below the surface of membranes (near the headgroup region of membranes [68,83,89]). Section 4.1 displays and discusses the approximate locations of CSL and 4PT spin-labels, along with their nitroxide moieties, within the lens lipid bilayer membrane. The detection of more α-crystallin binding with 4PT spin-label than CSL spin-label within the CM and NM implies that α-crystallin binding is mainly on the surface of these membranes. Interestingly, the detection of α-crystallin binding to the CMLC with the CSL spin-label implies that decreasing Chol content within the membrane increases the binding of α-crystallin below the surface. This also implies that with decreased Chol content within the membrane, α-crystallin penetrates the membrane. The MMSO for the CM, the CMLC and the NM reported in this paper are comparable with the MMSO values obtained for the individual and two-component lipid membranes [82,83,84,85] and the model of human and animal lens lipid membranes [86]. Furthermore, the MMSO for the CM, the CMLC and the NM reported agree with the result that ~10% of α-crystallin binds to PC vesicles [51]. 

The Chol/lipid mixing ratio in the two-year-old bovine CM and NM is 0.7 and 1.9 [69], respectively. With such a high Chol content in the bovine NM, Chol saturates the membrane forming immiscible cholesterol bilayer domains (CBDs), which coexist with the phospholipid cholesterol domain (PCD) [69,90]. However, due to low Chol content, the bovine CM does not contain CBDs [69,90]. The acyl chains of lipids become shorter and more saturated, with noteworthy changes in the lens lipid composition with age [57,63,72,91,92]. Between cortex and nucleus of clear lenses from various age groups and age-matched cataractous human lenses, these variations in lens lipid composition are observed [68,77,93]. Lens membrane SLs are roughly three to four times more saturated than PLs [74,80,81]. The bovine CM has a PC/SM molar ratio of 2 and the NM has a PC/SM molar ratio of 0.5, indicating remarkably low PC and higher SM content in the NM than in the CM [69]. Therefore, bovine NMs have a lower degree of unsaturation than CMs. Our previous studies show that Chol and CBDs inhibit the α-crystallin binding to membranes [84,85,86,87] and the SM membrane has higher MMSO than the 1-palmitoyl-2-oleoyl-*sn*-glycero-3-phosphatidylcholine (POPC) membrane [82,83]. A high SL and a lower PL content in the NM than in the CM may be the main reason why the NM has higher K_a_ than the CM. More generally, the larger MMSO for the NM should be due to the synergistic effects of a higher Chol content with CBDs, a higher SM/PC molar ratio and lipids with lower degrees of unsaturation in the NM than in the CM. The CMLC has larger MMSO than the CM, as shown in Figure 1A, because the decreased Chol content within the membrane increases the α-crystallin binding to the membrane, as reported in our previous model membrane studies [84,85,86,87].

The percentage of membrane surface occupied by α-crystallin plotted as a function of α-crystallin concentrations data were fitted using a one-site ligand binding model in GraphPad Prism (San Diego, CA, USA) to calculate the binding affinity (K_a_), as explained in our previous studies.

**Figure 2:**
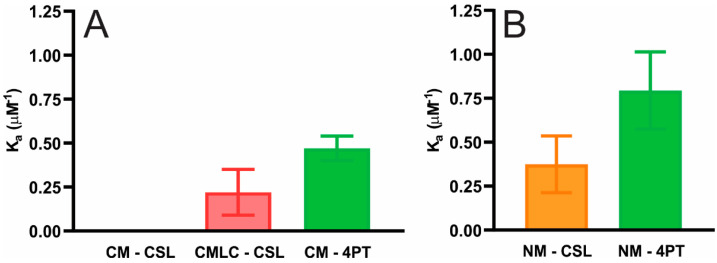
(**A**) Showing the binding affinity (K_a_) of α-crystallin to the bovine CMs with the CSL and 4PT spin-labels within these membranes and the CMLC with the CSL spin-label within the membrane. (**B**) Demonstrating the K_a_ of α-crystallin binding to the bovine NMs with the CSL and 4PT spin-labels within these membranes. The K_a_ below the surface of the CM detected by the CSL spin-label is zero, implying that the CSL spin-label does not detect α-crystallin binding to the CM. However, the K_a_ below the surface of the CMLC detected by the CSL spin-label within the membrane is non-zero, implying that decreasing Chol content within the membrane increases the strength of α-crystallin binding to membranes. The NMs have higher K_a_ than the CMs with both the CSL and 4PT spin-labels and the difference is statistically significant with *p* ≤ 0.05. Results are the mean ± standard deviation (σ) from at least three independent experiments.

The solid lines in Figure 1 are from fitting the data. The CSL and 4PT spin-labels detect the K_a_ of α-crystallin binding below and on the surface of membranes, respectively. Using the CSL spin-label, we estimated the K_a_ of α-crystallin binding below the surface of the CM, the CMLC and the NM to be 0, 0.22 ± 0.13 μM^−1^ and 0.47 ± 0.07 μM^−1^, respectively, as shown in Figure 2A. Using the 4PT spin-label, we estimated the K_a_ of α-crystallin binding on the surface of the CM and NM to be 0.37 ± 0.16 μM^−1^ and 0.79 ± 0.22 μM^−1^, respectively, as shown in Figure 2B. The differences between the K_a_ values below and on the surface of CM and NM are statistically significant with *p* ≤ 0.05. The difference between the K_a_ values below the surface of the CM and CMLC is statistically significant with *p* ≤ 0.05. With the CSL spin-label, the higher K_a_ for the CMLC compared to the CM represents that decreasing Chol content within the membrane causes α-crystallin to bind below the surface of the membrane strongly. With both the CSL and 4PT spin-labels, the higher K_a_ for the NM compared to the CM represents that α-crystallin binds more strongly to the NM than it does to the CM. Our previous study shows that a lower degree of unsaturation of PLs gives higher K_a_ for α-crystallin binding [84]. The NM has less degree of unsaturation of acyl chains than the CM, which may be the main reason the NM has higher K_a_ than the CM. More typically, the larger K_a_ for the NM should be due to the synergistic effects of a higher Chol content with CBDs, a higher SM/PC molar ratio and lipids with lower degrees of unsaturation in the NM than in the CM. Previously, using the fluorescence approach, the study of αA- and αB-crystallins binding to human CM and NM containing intrinsic membrane proteins also reported the higher binding capacities of these crystallins to NMs than CMs [58]. Similarly, using sucrose density gradient centrifugation, the higher binding of α-crystallin to the human lens NM than to the CM, where both the CM and NM include intrinsic membrane proteins, has been reported previously [41,43]. The K_a_ values for the CM and NM reported in this paper agree with the K_a_ values reported in our previous study for the individual and two-component lipid membranes [82,83], cholesterol-containing lipid membranes [85] and the model of human and animal lens lipid membranes [86]. The CM, the CMLC and the NM used in this study contain only total lipids (PLs, SLs and cholesterol) without intrinsic membrane proteins. It is likely that because of the intrinsic membrane proteins in alkali-washed lens plasma membranes, the K_a_ = 7.69 μM^−1^ reported by Mulders et al. [51] for the α-crystallin binding to plasma membranes is higher than the K_a_ reported in this study for the bovine CM, the CMLC and the NM. 

### 2.2. Mobility Below and on the Surface of the Bovine Cortical and Nuclear Membranes with the α-Crystallin Binding

**Figure 3:**
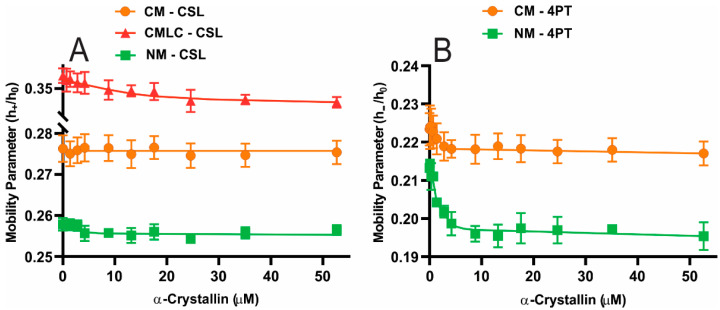
(**A**) The mobility parameter (h_+_/h_0_) profiles obtained at 37 °C using the CSL spin-labels within the bovine CM, the CMLC and the NM plotted as a function of α-crystallin concentration. (**B**) The mobility parameter (h_−_/h_0_) profiles obtained at 37 °C using the 4PT spin-labels within the bovine CM and NM derived from a single lens plotted as a function of α-crystallin concentration. The NM has less mobility parameter than the CM with both the CSL and 4PT spin-labels, implying that the NM below and on the surface is less mobile than the CM. With the CSL spin-label, the mobility parameter of the CMLC is higher than the CM, implying that the decreased Chol content within the CMLC increased the mobility parameter. For the CMLC with CSL spin-label, the CM with 4PT spin-label and the NMs with both CSL and 4PT spin-labels, the mobility parameter decreases with increased α-crystallin concentration, implying that the α-crystallin binding to these membranes decreases mobility below and on the surface of membranes. Results are the mean ± standard deviation (σ) from at least three independent experiments.

The mobility parameter of the NM is smaller than the CM for both the CSL and 4PT spin-labels within the membrane, as shown in Figure 3, suggesting that the NM below and on the surface is less mobile than the CM. The mobility parameter for CM and NM with CSL and 4PT spin-labels have statistically significant differences with a *p*-value ≤ 0.05. Interestingly, a less mobile NM below and on the surface has higher MMSO and K_a_ than the CM, showing that a less mobile NM below and on the surface can strongly bind a higher amount of α-crystallin than the CM. With the CSL spin-label, the mobility parameter of the CMLC is larger than the CM, as shown in Figure 3A, suggesting that the CMLC below the surface became more mobile due to decreased Chol content. The mobility parameter for the CM and the CMLC with CSL spin-label have statistically significant differences with a *p*-value ≤ 0.05. Our previous studies showed that the increased Chol content decreases the mobility parameter of the cholesterol-containing individual lipid membranes [84,85] and the model of human and animal lens lipid membranes [86] using the CSL spin-label within these membranes. Moreover, using the CSL spin-label, our previous studies show that the POPC membrane has a higher mobility parameter than the SM membrane [83,85] and the membranes with shorter acyl chain length and a lower degree of unsaturation of lipids have a smaller mobility parameter [84]. Moreover, lens membrane SLs are roughly three to four times more saturated than PLs [74,80,81]. Since the bovine NM have significantly higher Chol content with CBDs within the membrane [69], higher SM content and lower PC content [69] and a lower degree of unsaturation than the CM, the NM below and on the surface is less mobile than the CM. 

When the CSL spin-labels are within the membranes (Figure 3A), the mobility parameter of the CMLC and the NM decreases with increased α-crystallin concentrations, representing that the CMLC and the NM below the surface become less mobile with α-crystallin binding. Since the CSL spin-labels did not detect the α-crystallin binding to the CM, there is no significant change in the mobility parameter of the CM with the CSL spin-labels. When the 4PT spin-labels are within membranes (Figure 3B), the mobility parameter of both the CM and NM decreases significantly with increased α-crystallin concentrations. This indicates that the CM and NM on the surface become less mobile with α-crystallin binding. The total decrease in the mobility parameter and how rapid the mobility parameter decreases with α-crystallin binding are determined by the MMSO and K_a_. The higher the MMSO, the higher the total decrease in the mobility parameter and vice-versa. The higher the K_a_, the more rapid the decrease in the mobility parameter and vice-versa. Since the MMSO for the NM with the 4PT spin-label is higher than the CM, the total decrease in the mobility parameter of the NM is more than the CM. Since the K_a_ for NMs with the 4PT spin-label is higher than the CMs, the decrease in the mobility parameter of the NMs is more rapid than it is for the CMs. The observation of a significant decrease in mobility parameters of nuclear and CMs with the 4PT spin-labels than the CSL spin-labels also suggests that the binding of α-crystallin is likely on the surface of these membranes. Moreover, the observation of a significant decrease in the mobility parameter of the CMLC with CSL spin-label suggests that decreasing Chol content within the membrane increases the binding of α-crystallin below the surface of the membrane. Our previous EPR spin-labeling studies showed a decrease in the mobility parameter of individual and two-component lipid membranes [82,83], cholesterol-containing individual lipid membranes [85] and cholesterol-containing models of the human and animals lens lipid membranes [86] below the surface of these membranes after α-crystallin binding. Borchman and Tang [46] used a fluorescence approach to investigate the α-crystallin binding to bovine lens lipid vesicles and found that α-crystallin binding to vesicles made the lipid headgroups less mobile. Liang and Li et al. [56] performed fluorescence polarization measurements and reported that α-crystallin binding to bovine lens lipid membranes makes the lipid bilayers less mobile. Therefore, it is possible that the α-crystallin binding to the bovine CM and NM stabilizes membranes on the surface, as suggested by previous studies [46,56]. 

### 2.3. Order Below and on the Surface of the Bovine Cortical and Nuclear Membranes with the α-Crystallin Binding

**Figure 4:**
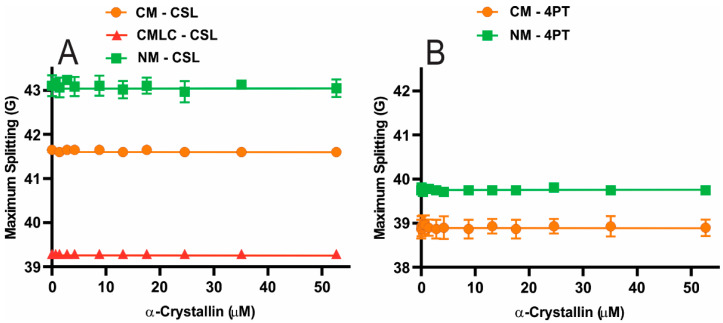
(**A**) The maximum splitting profiles obtained at 37 °C using the CSL spin-labels within the bovine CM, the CMLC and the NM plotted as a function of α-crystallin concentration. (**B**) The maximum splitting profiles obtained at 37 °C using the 4PT spin-labels within the bovine CM and NM derived from the total lipids extracted from a single lens plotted as a function of α-crystallin concentration. The smaller maximum splitting of the CM than the NM for the CSL and 4PT spin-labels implies that the CM below and on the surface is less ordered than the NM. With CSL spin-labels within the membranes, the maximum splitting of the CMLC is smaller than the CM. This implies that decreased Chol content within membranes decreases the order below the surface of membranes. Even with α-crystallin binding to the CM, the NM and the CMLC, the maximum splitting does not change significantly, implying that α-crystallin binding to these membranes does not significantly change the order below and on the surface. Results are the mean ± standard deviation (σ) from at least three independent experiments.

The maximum splitting of the NM is larger than the CM for both the CSL and 4PT spin-labels within these membranes, as shown in Figure 4, suggesting that the NM below and on the surface is more ordered than the CM. The maximum splitting values for CM and NM with CSL and 4PT spin-labels have statistically significant differences with a *p*-value ≤ 0.05. Interestingly, a more ordered and less mobile NM below and on the surface has higher MMSO and K_a_ than the CM, showing that a higher ordered and less mobile NM strongly binds a higher amount of α-crystallin than the CM. The maximum splitting of the CMLC is smaller than the CM with CSL spin-label in both membranes, as shown in Figure 3A, suggesting that the CM below the surface becomes less ordered with decreased Chol content. The maximum splitting for the CM and the CMLC with CSL spin-label have statistically significant differences with a *p*-value ≤ 0.05. Our previous studies showed that the increased Chol content increases the maximum splitting of the cholesterol-containing individual lipid membranes [84,85] and the model of human and animal lens lipid membranes [86] using the CSL spin-label within these membranes. Moreover, using the CSL spin-label, our previous studies showed that the POPC membrane has a lower maximum splitting than the SM membrane [83,85] and the membrane with a shorter acyl chain length and a lower degree of unsaturation of lipids has a larger maximum splitting [84]. Moreover, lens membrane SLs are roughly three to four times more saturated than PLs [74,80,81]. Since the bovine NM has significantly higher Chol content with CBDs within the membrane, higher SM content and lower PC content [69] and a lower degree of unsaturation of lipids than the CM, the NM below and on the surface is more ordered than the CM. PC content decreases and SM content increases in an aged lens membrane [72,91]. The acyl chains of lipids become shorter and more saturated, with noteworthy changes in the lens lipid composition with age [57,63,72,91,92]. Between cortex and nucleus of clear lenses from various age groups and age-matched cataractous human lenses, these variations in lens lipid composition are observed [68,77,93]. Our observations of the higher order below and on the surface of the NM than the CM supports the increased order of an aged lens membrane [72,91]. 

Even with a significant binding of α-crystallin to CM and NM detected by the 4PT spin-label and the CMLC and NM detected by the CSL spin-label, the maximum splitting of these membranes does not change significantly with α-crystallin concentrations. These results show that the order below and on the surface of membranes does not change significantly with α-crystallin binding. Similar to this study, the maximum splitting of the individual and two-component lipid membranes [82,83,84], cholesterol-containing individual lipid membranes [84,85] and cholesterol-containing models of the human and animals lens lipid membranes [86] did not change significantly with α-crystallin binding to these membranes in our previous studies, except for the SM and SM/POPE membranes [83]. 

### 2.4. Hydrophobicity Below and on the Surface of the Bovine Cortical and Nuclear Membranes with the α-Crystallin Binding

**Figure 5:**
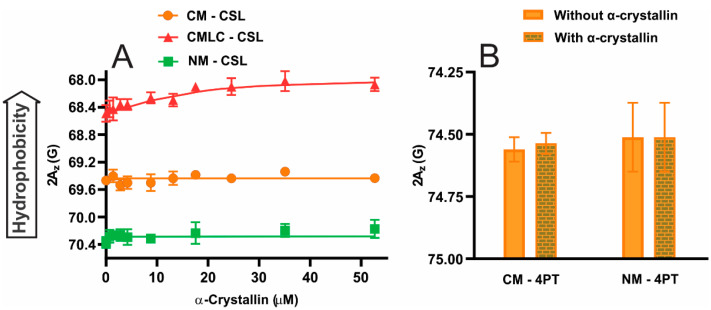
(**A**) Hydrophobicity (2A_z_) below the surface of the bovine CM, the CMLC and the NM with the CSL spin-label within these membranes is plotted as a function of α-crystallin concentration. (**B**) Hydrophobicity on the surface of the bovine CM and NM with the 4PT spin-label is shown with 52.6 μM α-crystallin and without α-crystallin. Figure 1 of our previous study [86] shows the method for 2A_z_ measurement. A decrease in 2A_z_ indicates an increase in hydrophobicity [89,94,96,97,98]. The hydrophobicity of the CM and NM with CSL and 4PT spin-labels does not change significantly, even with α-crystallin binding to these membranes. However, with the CSL spin-label, the hydrophobicity of the CMLC increases with increased α-crystallin concentration. This implies that decreased Chol content within the CMLC allows the binding of α-crystallin below the surface of this membrane, increasing the hydrophobicity below the surface of this membrane. Results are the mean ± standard deviation (σ) from at least three independent experiments.

The hydrophobicity of the CM with the CSL spin-label is larger than the NM (Figure 5A), implying that the hydrophobicity below the surface of the CM is larger than the NM. Furthermore, with the CSL spin-label, the hydrophobicity of the CMLC is larger than the CM (Figure 5A), implying the hydrophobicity below the surface of the CMLC is larger than the CM. The hydrophobicity below the surface of the CM, the CMLC and the NM have a statistically significant difference with *p* ≤ 0.05. Our previous EPR study [84] shows that the hydrophobicity below the surface of the membrane does not change significantly with a change in acyl chain length and degree of unsaturation of lipids in membranes. However, our previous EPR studies show that the hydrophobicity below the surface of the cholesterol-containing individual lipid membranes [84,85] and models of the human and animal lens membranes [86] decrease with increased Chol content. Our previous EPR studies also showed that increased Chol content decreases hydrophobicity below the surface of Chol/SM [94], Chol/POPC [89] and cholesterol/1-palmitoyl-2-oleoyl-sn-glycero-3-phosphatidylserine (Chol/POPS) [95] multilamellar vesicles. The increased Chol content separates the polar headgroups of lipids in membranes and increases the water penetration below the surface of membranes, decreasing the hydrophobicity (increasing the polarity) [96]. Therefore, the higher Chol content with CBDs within the NM than in the CM [69] should be the main reason that hydrophobicity below the surface of the NM is smaller than below the surface of the CM. The higher Chol content within the CM than in the CMLC should be why hydrophobicity below the surface of the CM is smaller than below the surface of the CMLC. Similar hydrophobicity for CM and NM with the 4PT spin-label represents similar hydrophobicity on the surface of these membranes (Figure 5B). This shows that the noteworthy difference in lipid and Chol composition between the CM and NM, with significantly higher Chol content with CBDs within the NM [69], does not significantly change the hydrophobicity on the surface of these membranes. The hydrophobicity of the CM and NM detected by the CSL spin-label (Figure 5A) is much higher than the 4PT spin-label (Figure 5B). This is because the water penetration below the surface of the CM and NM, where the nitroxide moiety of the CSL spin-label resides, is less than on the surface of these membranes, where the nitroxide moiety of the 4PT spin-label is located. 

The hydrophobicity of the CM and NM with CSL spin-label does not change significantly with increased α-crystallin concentration (Figure 5A), showing no change in hydrophobicity below the surface of these membranes. However, the hydrophobicity of the CMLC with CSL spin-label within the membrane increases significantly with increased α-crystallin concentration (Figure 5A), showing increased hydrophobicity below the surface of this membrane. No change in hydrophobicity for the CM with the CSL spin-label is expected because the binding of α-crystallin to the CMs is not detected by the CSL spin-label (see Figure 1A). With increased α-crystallin concentration, no significant change in hydrophobicity for the NM with the CSL spin-label is attributed to the small amount of α-crystallin binding to the NM detected by the CSL spin-label (See Figure 1A). With increased α-crystallin concentration, the significant increase in hydrophobicity for the CMLC with the CSL spin-label within the membrane is attributed to the significant amount of α-crystallin binding to this membrane detected by the CSL spin-label (See Figure 1A). This result shows that decreased Chol content within the CMLC increases the binding of α-crystallin to the membrane and increases the hydrophobicity below the surface of the membrane, indicating that α-crystallin binding forms the hydrophobic barrier for the passage of polar and ionic molecules, supporting the barrier hypothesis in cataract formation. Our previous EPR studies suggested the hydrophobic binding of α-crystallin to membranes [83,85,86], where α-crystallin’s hydrophobic residues on the surface bind to membranes [85,86]. Even with lower hydrophobicity below the surface of the NM than the CM, the CSL spin-label detects a small amount of binding of α-crystallin to the NM and no binding of α-crystallin to the CM. This result suggests that the order and mobility below the surface of membranes, with noteworthy differences in lipid and Chol composition, strongly modulate the likely hydrophobic binding of α-crystallin to membranes. Interestingly, even with similar hydrophobicity on the surface of the CM and NM detected by 4PT spin-labels (Figure 5B), the α-crystallin binding to the NM is much larger, with significantly larger MMSO and K_a_, than the CM. This result further supports the claim that the order and mobility of the membranes, with noteworthy differences in lipid and Chol composition, strongly modulate the likely hydrophobic binding of α-crystallin to membranes. Even with the significantly large binding of α-crystallin to the CM and NM detected by the 4PT spin-label (Figure 1B), the hydrophobicity of these membranes does not change significantly in the presence of 52.6 μM α-crystallin (Figure 5B). This shows that the hydrophobicity on the surface of the CM and NM does not change significantly with the α-crystallin binding. This data also supports our claim that the α-crystallin binding is mainly on the surface of the CM and NM. Such binding of α-crystallin to the membrane surface does not form a hydrophobic barrier and may stabilize the membrane, as suggested by the previous studies [46,56].

## 3. Discussion

The bovine NM has higher Chol content [69], a higher SM/PC molar ratio [69] and shorter acyl chains with a lower degree of unsaturation than the CM [57]. Due to such noteworthy differences in lipid and Chol composition, the α-crystallin binding to the bovine NM is higher with larger MMSO and K_a_ than the CM. The larger MMSO and K_a_ for the NM compared to the CM correlates with low mobility and high order below and on the surface of the NM compared to the CM. The Chol content in the human lens membrane decreases with cataracts [76,77]. Our results show that the decreased Chol content within the CM increases the binding of α-crystallin below the surface of the membrane. Moreover, the study reported in this paper shows that the α-crystallin binding to the bovine CM and NM decreases the mobility with no significant change in order and hydrophobicity below and on the surface of membranes. However, α-crystallin binding to the CMLC, which mimics the decreased Chol content in the cataractous lens membranes [76,77], decreases mobility, increases hydrophobicity and does not significantly change the order below the surface of the membrane. This result shows that decreased Chol content within the CM increases hydrophobicity below the surface of the membrane, increases the binding of α-crystallin to the membrane and forms the hydrophobic barrier for the passage of polar and ionic molecules, supporting the barrier hypothesis in cataract formation. Results also show that the hydrophobicity of the NM below the surface is less than the CM, mainly because the NM has a higher Chol content, which separates the polar headgroups increasing the water penetration below the surface [96]. However, the hydrophobicity values of the nuclear and CMs on the surface are almost equal because, despite the noteworthy differences in lipid and Chol composition between these membranes, the water accessibility on the surface of these membranes is similar. Our data represent that the order and mobility of the CM and NM, with noteworthy differences in lipid and Chol composition, strongly modulate the likely hydrophobic binding of α-crystallin to these membranes. Our study provides a deeper understanding of α-crystallin binding to the bovine CM and NM with noteworthy differences in lipid and Chol composition. In addition, the study reported in this paper shows the feasibility of such experiments using the total lipids extracted from a single lens cortex and nucleus of a human. The human lens lipid composition changes not only because of age but also because of medical history. Moreover, a human’s left and right lenses may have very different lipid and Chol compositions, as only one eye of a human may have a cataract. Therefore, experiments with the CM and NM derived from the total lipids extracted from a single lens of a human are very important and the study reported in this paper opens a clear avenue in that direction.

We used native bovine eye lens α-crystallin, which consists of αA- and αB-crystallin in a 3:1 molar ratio [7]. Srinivas et al. [99] reported that αA: αB = 3:1 heteropolymer and αA homopolymer does not precipitate below their unfolding temperature between 58 to 61 °C. The EPR spectra of the CSL and 4PT spin-labels within the bovine CM and NM are typical lipid bilayer spectra, with changes only due to the difference in spin-labels and the lipid and Chol composition. Section 4.4 displays and discusses typical lipid bilayer spectra of the 4PT spin-label within the NM with 52.6 μM α-crystallin and without α-crystallin. As in our previous studies for individual lipid membranes, cholesterol-containing individual lipid membranes and the model of human and animal lens lipid membranes [82,83,85,86], no significant change in the EPR spectra has been observed for bovine CM and NM with incubation at 37 °C for 16 h and without incubation. These observations ensure that our samples, including α-crystallin and membranes, are stable during the experiment.

Our results show that the nitroxide moiety of the CSL spin-label located below the membrane surface (near the headgroup regions of the membrane [68,83,89]) monitors deeper α-crystallin binding to the membrane and the nitroxide moiety of the 4PT spin-label located on the membrane surface (in the aqueous phase close to the membrane surface [88]) monitors the surficial binding of α-crystallin to the membrane. Our previous studies show that Chol and CBDs inhibit the binding of α-crystallin to membranes [84,85,86,87]. As the nitroxide moiety of the 4PT spin-label resides on the membrane surface, it is beneficial to use in the study of α-crystallin binding to high cholesterol-containing membranes, where the binding is minimal. Since the human lens membrane has exceptionally high Chol content with a Chol/lipid molar ratio as high as 4.4 in the nucleus [67,68,70,71], the approach of using both CSL and 4PT spin-labels might provide deeper insights into α-crystallin membrane binding studies.

The detection of more α-crystallin binding to the CM and NM with the 4PT spin-label, with a significant decrease in the mobility on the surface of these membranes, than the CSL spin-label within these membranes implies that α-crystallin binds mainly on the surface of these membranes. Such surface binding does not form a hydrophobic barrier on the surface of these membranes (see Figure 5B) and is likely to make the lens lipid bilayer more stable. With decreased Chol content within the CM and NM, α-crystallin would penetrate the membrane forming a hydrophobic barrier, likely making the lipid bilayer unstable. We have decreased the Chol content within the CMs with the CSL spin-label and observed an increase in hydrophobicity below the surface of membranes with α-crystallin binding (see Figure 5A). Such an increase in the hydrophobicity due to α-crystallin binding forms a hydrophobic barrier below the surface of the membrane, which likely disrupts lens membrane homeostasis and promotes cataract formation. Our previous study [84] using CSL spin-labels also shows that the hydrophobicity of individual 1-stearoyl-2-oleoyl-sn-glycero-3-phosphocholine (SOPC), cholesterol-containing SOPC and the models of human and animal lens lipid membranes increases significantly with α-crystallin binding, forming a hydrophobic barrier below the surface of these membranes. Therefore, Chol in the lens membrane not only inhibits α-crystallin binding to lens membranes [84,85,86] but also prevents the formation of a hydrophobic barrier, possibly playing a significant role in maintaining lens transparency.

The bovine lenses we used are clear (no cataract); however, there is a significant surficial binding of α-crystallin to both the CM and NM. Our results show that, even though there is no cataract, α-crystallin can bind with the membranes implying that α-crystallin binding to membranes may not be the only cause of cataract formation. Indeed, such surficial binding of α-crystallin to membranes in clear and young lenses may play a beneficial role in membrane stability. However, age-related changes within membranes (lipid and cholesterol oxidation [100,101,102,103,104,105], changes in lipid composition [61,62,68,70,72,73,74,75] and changes in the saturation level of the lipids [57,63,72,91,92]) and in the crystallin proteins (mutations [14,15,16,17] and post-translational modifications [18,19,20,21,22,106,107,108,109,110,111]) may denature proteins, significantly decrease the chaperone-like activity of α-crystallin and initiate the excessive accumulation of HMWC on the lens membranes. Such excessive accumulation of HMWC blocks the flow of water and small metabolites between the lens membranes and forms a barrier, as described earlier in the old and cataractous lenses [37,70,112]. The research reported in this paper clearly showed that the lipid compositional difference between the CM and NM of a single young bovine lens strongly modulates the binding of α-crystallin to these membranes. Additionally, this research showed that the decreased Chol content within the membrane increases the binding of α-crystallin below the surface of the membrane and increases hydrophobicity, forming the hydrophobic barrier to the passage of polar and ionic molecules and supporting the barrier hypothesis in cataract formation. In future research, it would be even more beneficial to compare the α-crystallin-membrane binding in young and old lenses as well as age-matched cataractous lenses to better understand the mechanism of α-crystallin binding to membranes and cataract formation.

The bovine CM, the CMLC and the NM derived from the total lipids extracted from a single lens cortex and nucleus used in this study do not include intrinsic membrane proteins, such as MIP26 [113,114,115] and connexins [116]. Studies involving MIP26 and isolated crystallins revealed that α-crystallin binds to MIP26 [51,56,117]. Some studies, however, assert that α-crystallin mostly binds to lipids [37,46,47,50,57,59]. Using the PL vesicles with reconstituted MIP26, Mulders et al. [51] demonstrated that the presence of MIP26 strongly affects the α-crystallin binding to vesicles. To better understand the effect of intrinsic membrane proteins on α-crystallin binding to lens lipid membranes, further studies in this direction are needed. 

The research performed in different laboratories on blocking or preventing cataract formation is focused on the development of cholesterol derivate compounds (CDCs) such as lanosterol and 25-hydroxycholesterol [118,119], reported to be effective for decreasing α-crystallin aggregation and preventing cataract formation [118,119]; however, these findings disagree with results from other laboratories [105,120,121]. It is reported that lanosterol reverses α-crystallin aggregation and maintains lens transparency in dogs’ in-vivo and in dissected rabbits’ cataractous lenses in-vitro [118]. Similarly, based on the in-vitro, in-vivo and ex-vivo studies on mice [119], it has been reported that 25-hydroxycholesterol binds to a specific region of α-crystallin restoring solubility of this protein and partially reversing its aggregation and cataract formation [119]. In contrast to these findings, it has been reported that 25-hydroxycholesterol presence is connected with cataracts in human lenses [105]. In addition, it has been reported that the culture of lanosterol with the lens nuclei from cataractous human lenses failed to reverse protein aggregation and restore lens nuclei transparency [120]. The study was performed using whole lens culture and human lens binding studies provided no evidence that CDCs like lanosterol or 25-hydroxycholesterol bind aggregated protein and reverse cataracts [121]. The approach to developing CDCs in preventing cataract development focused on the interaction of the CDCs to α-crystallin to prevent α-crystallin aggregation [118,119]. However, a longitudinal clinical study [45] performed with 45 patients (66 eyes) aged 34–79 years using dynamic light scattering (DLS) shows that the higher levels of membrane-bound α-crystallin, with a corresponding decrease in the unbound α-crystallin concentration in the lens cytoplasm, are associated with nuclear cataract formation and progression [45]. Results presented in this manuscript on the native lens lipid membranes and our previous studies on model membranes [84,86] clearly show that increased Chol content decreases the hydrophobicity below the surface of membranes and inhibits α-crystallin binding to membranes, likely preventing cataract formation. Thus, determining if the CDCs could be incorporated into the membrane that significantly inhibits α-crystallin binding below the membrane surface may be crucial in preventing the early stage of cataract development. Thus, developing CDCs and incorporating these CDCs into the lens membrane that significantly decreases hydrophobicity below the membrane surface may be crucial in inhibiting α-crystallin binding below the membrane surface and preventing the formation of the hydrophobic barrier, likely protecting against cataract formation and progression.

## 4. Materials and Methods

### 4.1. Materials

Fresh eye lenses of an approximately two-year-old bovine were obtained from Pel-Freez, LLC (Rogers, AR, USA) on the day of slaughter and immediately stored at −80 °C until total lipids isolation was performed. The total lipids from the cortex and nucleus of a single lens were extracted using a monophasic extraction protocol [121], as explained in detail in Section 4.2. Cholesterol analog cholestane spin-label (CSL), native bovine eye lens α-crystallin (C4163), HEPES and sodium chloride (NaCl) were obtained from Sigma Aldrich (St. Louis, MO, USA). 1-palmitoyl-2-oleoyl-sn-glycero-3-phosphatidylcholine (POPC), 1-palmitoyl-2-oleoyl-sn-glycero-3-phosphoethanolamine (POPE), 1-palmitoyl-2-oleoyl-sn-glycero-3-phosphatidylserine (POPS), sphingomyelin (SM) and 4-palmitamido-TEMPO (4PT) spin-label were obtained from Avanti Polar Lipids, Inc. (Alabaster, AL, USA). POPC, POPS, POPE and SM were obtained in chloroform, CSL and 4PT spin-labels were dissolved in chloroform and native bovine lens α-crystallin was dissolved in HEPES buffer (10 mM HEPES, 100 mM NaCl, pH = 7.4). α-Crystallin was used without further purification. 

**Figure 6:**
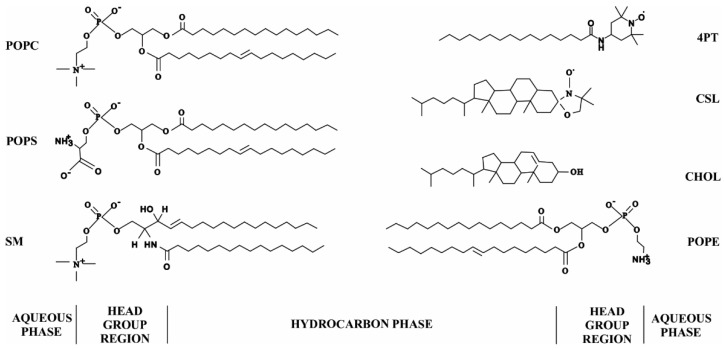
The molecular structure of the major lipids of the eye lens, such as phospholipids (1-palmitoyl-2-oleoyl-sn-glycero-3-phosphatidylcholine (POPC), 1-palmitoyl-2-oleoyl-sn-glycero-3-phosphoethanolamine (POPE) and 1-palmitoyl-2-oleoyl-sn-glycero-3-phosphatidylserine (POPS)), sphingomyelin (SM) and cholesterol (Chol). The molecular structures of spin-labels (cholesterol analog spin-label (CSL) and 4-palmitamido-TEMPO (4PT)) used in this study are also shown. The estimated location of each molecule on the lipid bilayer membrane is displayed. The nitroxide moieties of CSL and 4PT spin-labels reside below the surface of the membrane (below the membrane’s headgroup regions [68,83,89]) and on the surface of the membrane (in the aqueous phase close to the membrane surface [88]), respectively.

SLs, i.e., SM and dihydro-SM (DHSM), and PLs, i.e., phosphatidylcholine (PC), phosphatidylserine (PS) and phosphatidylethanolamine (PE), make up the majority of the lipids in the eye lens membrane [63]. PLs are made up of two fatty acids “tails” and a “head” group that designates the kind of PL, such as PC, PE and PS. The eye lens’ fiber cell membranes primarily include palmitic (16:0, P) and oleic (18:1-cis, O) fatty acid tails [63,67,72,122]. Lens membranes of different species have different lipid and Chol compositions [63,65,123,124,125]. In contrast to PC, which predominates in short-lived animals, SLs (mostly DHSM) are predominant in humans [63]. 1-palmitoyl-2-oleoyl-sn-glycero-3-phosphatidylcholine (POPC), 1-palmitoyl-2-oleoyl-sn-glycero-3-phosphatidylserine (POPS), 1-palmitoyl-2-oleoyl-sn-glycero-3-phosphoethanolamine (POPE) and SM are commonly used lipids to mimic the lipid composition of the lens membrane. Figure 6 shows the molecular structures of the POPC, POPS, POPE and SM, as well as that of Chol and spin-labels (CSL and 4PT). The approximate location of each molecule on the lipid bilayer membrane is displayed. The nitroxide moieties of CSL and 4PT spin-labels reside below the surface of the membrane (below the membrane’s headgroup regions [68,83,89]) and on the surface of the membrane (in the aqueous phase close to the membrane surface [88]), respectively.

### 4.2. Isolation of Total Lipids from the Single Lens Cortex and Nucleus of a Bovine

A single lens of an approximately two-year-old bovine was taken out of a −80 °C freezer and allowed to defrost at room temperature. The cortex and nucleus from a single lens were separated based on differences in tissue consistency [126,127]. Total lipids from cortical and nuclear fractions were isolated separately based on the minor modifications of a monophasic extraction protocol developed by Byrdwell et al. [128]. The cortical and nuclear tissues were transferred into separate glass centrifuge tubes containing 2 mL of methanol, homogenized separately using a glass Dounce homogenizer, and 13 mL of methanol was added to each glass tube. The cortical and nuclear tissues were further homogenized separately using a probe-tip sonicator (Fisher Scientific, Model 550, Waltham, WA, USA) three to four times for 15 s each time, with a 30 s cooling period in ice between sonication cycles. The homogenized solutions were centrifuged at 4 °C for 1 h at 5000 rpm using an Avanti J26S XP centrifuge and JA-25.50 rotor. The supernatant from each solution was decanted into glass beakers, leaving a smaller layer of supernatant in the centrifuge tubes to avoid disrupting the pellets. The glass beakers containing supernatants were placed on the hot plate (Thermo Scientific) and methanol in each beaker was evaporated at 60 °C using the controlled stream of N_2_-gas from the top. 10 mL of hexane and isopropanol (2:1 *v*/*v*) solutions were added to each beaker containing dry lipid films. Glass Dounce homogenizers were gently used to dissolve lipid films into hexane and isopropanol solutions. Then, solutions were transferred into glass centrifuge tubes and gently sonicated with a probe-tip sonicator for 15 s. The solutions were then centrifuged at 4 °C for 1 h at 5000 rpm. The supernatant containing total lipids from each tube was decanted into the fresh beaker, leaving a smaller layer of supernatant in the tubes to avoid disrupting the pellets. The hexane and isopropanol solutions were evaporated at 60 °C using the controlled stream of N_2_-gas from the top. When hexane and isopropanol solutions were evaporated to 2 mL, solutions were transferred to fresh glass centrifuge tubes, an additional 3 mL of hexane and isopropanol (2:1 *v*/*v*) was added to each tube and tubes were centrifuged again at 4 °C for 1 h at 5000 rpm to remove any remaining impurities. The supernatant from each tube was transferred into weighted small glass tubes and solutions were evaporated at 60 °C using the controlled stream of N_2_-gas from the top. The remaining traces of hexane and isopropanol were evaporated in a vacuum overnight. The weight of each glass tube with lipid films was measured and the total lipid from the cortex and nucleus of a single bovine lens was estimated. The total cortical and nuclear lipids isolated from a single bovine lens were estimated to be ~3.1 mg and ~1.9 mg, respectively. The cortical and nuclear lipids were dissolved in chloroform, maintaining 0.5 mg/mL of total lipids and were stored at −20 °C.

### 4.3. Preparation of the Bovine Cortical and Nuclear Unilamellar Vesicles (UVs)

The total lipids extracted from the cortex and nucleus of a single bovine lens in chloroform were mixed with chloroform solutions of the CSL and 4PT spin-labels separately in glass tubes to prepare the CM and NM. Concentrations of the CSL and 4PT spin-labels with respect to total lipids in CM and NM were maintained at 1 mol% and 2 mol%, respectively. The Chol/lipid mixing ratio in the two-year-old bovine CM and NM is 0.7 and 1.9 [69], respectively. The Chol content in the human lens membrane decreases with cataracts [76,77]. To mimic the decreased Chol content in the cataractous lens membrane, we prepared the CMLC by decreasing the Chol content in the CM making the Chol/lipid mixing ratio of 0.1. For this, we mixed chloroform solutions of total lipids extracted from the cortex of a single bovine lens and the appropriate amounts of lipid mixtures of POPC, POPS, POPE and SM that resembles the bovine lens lipid composition [63]. The CMLC includes 1 mol% CSL spin-label within the membrane. Chloroform was evaporated from the ~1 to 4 mL of chloroform solutions containing total lipids and spin-labels using the controlled flow of N_2_-gas and the final volume of solutions was maintained to ~75 μL. Approximately 360 μL of warm (~50 °C) buffer (3.6 mM HEPES, 36 mM NaCl, pH = 7.4) was added to ~75 μL chloroform mixture. The rapid solvent exchange (RSE) method [129,130,131] was used to prepare the large multilamellar vesicles (LMVs), as described previously [132,133]. Briefly, the glass tube containing warm buffer and chloroform mixture was placed on the vortexer and connected to the sample manifold of the RSE equipment. The manifold valve was promptly opened to a vacuum system set at ~25 Torr once the vortexer was activated. After ~10 min, the sample was flushed with argon flow, the vortexer was turned off, the manifold was vented and the sample tube was withdrawn. A reduction in the final buffer volume containing LMVs to ~150 μL demonstrated that all the chloroform and some solvent had been evaporated. 

Uni-lamellar vesicles (UVs) were prepared by the sonication of LMVs, using a probe-tip sonicator (Fisher Scientific, Model 550), 25 times for 10 s sonication cycles followed by 15 s cooling in ice and 30 s heating in ~50–60 °C water bath. The final concentration of total lipid in UVs in 130 μL HEPES buffer (10 mM HEPES, 100 mM NaCl, pH = 7.4) was maintained at 24 mM. For brevity, we assumed the average molecular weight of the lipid in the extracted total lipid to be 750 Da and the molecular weight of the Chol to be 386 Da. A fixed concentration (11.4 mM) of total lipid in membranes was mixed with varying concentrations (0–52.6 μM) of α-crystallin in HEPES buffer (10 mM HEPES, 100 mM NaCl, pH = 7.4). The mixtures were incubated at 37 °C for 16 h in a Corning LSE benchtop shaking incubator (Corning, NY, USA) with shaking at 150 rpm. With incubation at 37 °C, α-crystallin binding to membranes saturates at ~8 h [82].

### 4.4. The Electron Paramagnetic Resonance (EPR) Spin-Labeling Method to Investigate the α-Crystallin Binding to the Bovine Cortical and Nuclear Membranes Derived from a Single Lens

Incubated samples were filled into a gas-permeable methyl-pentene polymer (TPX) [134], having a 1.0 mm internal diameter. The continuous wave (CW) electron paramagnetic resonance (EPR) measurements were performed at 37 °C and about −165 °C using an X-band Brucker ELEXSYS 500 spectrometer connected with accessories to control the temperature. N_2_-gas was used to completely deoxygenate the samples and maintain the temperature at 37 °C. At 37 °C and the EPR spectra were taken with a modulation amplitude of 1.0 G and incident microwave power of 8.0 mW.

**Figure 7:**
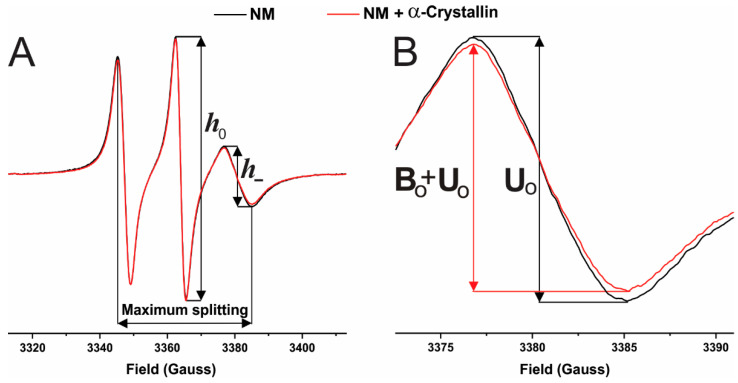
(**A**) The red and black spectra are the representative electron paramagnetic resonance (EPR) spectra of the 4PT in the bovine nuclear lens lipid membrane derived from a single lens with 52.6 μM α-crystallin and without α-crystallin, respectively. The ratio of peak to peak height of the high field line (h_−_) and the central line (h_0_) of the EPR spectra gives the mobility parameter (i.e., h_−_/h_0_) and the horizontal distance between the low field line and the high field line of the EPR spectra gives the maximum splitting. (**B**) It shows the zoomed high field lines of the spectra displayed in (**A**). The unbound ($U_{0}$) and unbound plus bound ($U_{0}$ + $B_{0}$) contributions are used to calculate the percentage of membrane surface occupied (MSO) by α-crystallin and the binding affinity (K_a_).

The representative normalized EPR spectra, normalized with respect to the central EPR line’s peak to peak intensity, for 4PT spin-labels in the NMs without α-crystallin (black) and with 52.6 μM α-crystallin (red) are shown in Figure 7A. Figure 7B, which shows the zoomed high field EPR lines of the spectra in Figure 7A, displays that α-crystallin binding to the NM decreases the peak to peak intensity of the high field EPR line. For 4PT spin-labels in the CM and NM in this study, the high filed line of the EPR spectra without α-crystallin was used as a control or unbound contribution ($U_{0}$) and the high filed line of the EPR spectra with various α-crystallin concentrations were used as unbound plus bound ($U_{0}$ *+* $B_{0}$) contribution, as represented in Figure 7B. Then, the percentage of 4PT spin-labels on the outer surface of the membrane affected due to the α-crystallin binding is measured using a similar method as used earlier for CSL spin-label in membranes [82,83,85] as:

(1) $$\%\;4\mathrm{PT}\;\mathrm{spin}\;\mathrm{labels}\;\mathrm{affected}=\left\{\frac{U_{0}-\left(U_{0}+B_{0}\right)}{U_{0}}\right\}\;100\%$$

DLS measurements on a DynaPro instrument (Wyatt Technology Corp., Santa Barbara, CA, USA) using regularization methods (Dynamics software, version 7) gave the size of the cortical and nuclear uni-lamellar vesicles to be ~120 nm. Based on that, ~52% and ~48% of the 4PT molecules are on the outer and inner surfaces of the membrane, respectively. Since the 4PT spin-labels that are on the outer surface are only affected by the binding of α-crystallin, the corrected percentage of 4PT spin label affected or the percentage of membrane surface occupied (MSO) by α-crystallin is estimated as:

(2) $$\%\;\mathrm{membrane}\;\mathrm{surface}\;\mathrm{occupied}\;(\mathrm{MSO})=(\%\;4\mathrm{PT}\;\mathrm{spin}\;\mathrm{labels}\;\mathrm{affected})\;\left(\frac{100}{52}\right)$$

The percentage of membrane surface occupied (MSO) by α-crystallin plotted as a function of α-crystallin concentrations data were fitted using a one-site ligand binding model in GraphPad Prism (San Diego, CA, USA) to calculate the binding affinity (K_a_), as explained in our previous studies [82,83,85].

With 4PT spin-labels within the CM and NM, we used the same procedure as described previously [135,136,137] for calculation of the mobility parameter with 1-palmitoyl-2-oleoyl-sn-glycero-3-phospho(tempo)choline (T-PC) spin-labels within membranes. Using the 4PT spin-labels within membranes, the ratio of peak to peak intensity of the high field and the central field EPR line (i.e., h_−_/h_0_) gives the mobility parameter. Figure 7A shows h_-_ and h_0_. The 4PT spin-label molecule’s motional freedom on the surface of the membrane increases when the mobility parameter (h_−_/h_0_) increases [135,136,137]. The horizontal distance between the low and high field EPR lines gives the maximum splitting using the 4PT spin-labels within membranes (see Figure 7A).

With CSL spin-labels within the CM, the CMLC and the NM, we calculated the MSO and K_a_ of α-crystallin binding to membranes based on the procedure described in our previous studies, where appropriate correction factor based on vesicle size is used. DLS measurements gave the radius of the CM and NM as ~120 nm and the CMLC as ~33 nm. Based on that, ~52% of the CSL molecules are on the outer surface of the CM and NM and ~56% of the CSL molecules are on the outer surfaces of the CMLC. Therefore, a correction factor of 100/52 is used to estimate the corrected % CSL spin-label affected or the MSO for CM and NM and a correction factor of 100/56 is used to estimate the corrected % CSL spin-label affected or the MSO for the CMLC, as performed earlier in our previous studies [82,83,85,86].

With CSL spin-labels within the CM, the CMLC and the NM, we used the same procedure as described in our previous studies [82,83,85,86] for calculating the physical properties of membranes (mobility parameter and maximum splitting). Briefly, using CSL spin-labels within membranes, the ratio of peak to peak intensity of the low field and the central field EPR line (i.e., h_+_/h_0_) gives the mobility parameter and the horizontal distance between the low field and the high field EPR line gives the maximum splitting [82,83,85,86]. The mobility parameter provides information regarding the orientational and rotational dynamics of the spin-labels in membranes [138]. The maximum splitting provides the amplitude of the wobbling motion of the long axes of spin-labels in membranes and is related to the order parameter [89,98,139]. 

The z-component of the hyperfine interaction tensor (A_z_) for CSL and 4PT spin-labels in the bovine cortical, the CMLC and the NM was measured from the EPR spectra recorded with a modulation amplitude of 2.0 G and an incident microwave power of 2.0 mW for samples frozen at about −165 °C [89,94,96,97,98]. Liquid nitrogen was used to maintain the temperature at about −165 °C. As shown in Figure 1 of our previous study [86], the horizontal distance between the low field line and the high field line of the EPR spectra of samples taken at about −165 °C gives the 2A_z_, a measure of hydrophobicity [89,94,96,97,98]. 

### 4.5. Statistics

All results are presented as mean ± standard deviation (σ) with at least three independent experiments. We evaluated the statistically significant differences in MMSO, K_a_, mobility parameter, maximum splitting and hydrophobicity values using the Student’s *t*-test with a *p* ≤ 0.05.

## Data Availability

Not applicable.

## Abbreviations

EPR	electron paramagnetic resonance
CM	cortical membrane
NM	nuclear membrane
CMLC	cortical membrane with low cholesterol
MSO	percentage of membrane surface occupied
MMSO	maximum percentage of membrane surface occupied
CSL	cholesterol analog cholestane spin-label
4PT	4-palmitamido-TEMPO
PL	phospholipid
SL	sphingolipid
Chol	cholesterol
